# Functional R2R3-MYB transcription factor *NsMYB1*, regulating anthocyanin biosynthesis, was relative to the fruit color differentiation in *Nitraria sibirica* Pall.

**DOI:** 10.1186/s12870-022-03561-5

**Published:** 2022-04-09

**Authors:** Xuemei Bao, Yuan Zong, Na Hu, Shiming Li, Baolong Liu, Honglun Wang

**Affiliations:** 1 Qinghai Provincial Key Laboratory of Tibetan Medicine Research and CAS Key Laboratory of Tibetan Medicine Research, Northwest Institute of Plateau Biology, Xining, 810008 China; 2 College of Education, Qinghai Normal University, Xining, 810008 China; 3 University of Chinese Academy of Sciences, Beijing, 100049 China; 4 Key Laboratory of Adaptation and Evolution of Plateau Biota (AEPB), Northwest Institute of Plateau Biology, Chinese Academy of Sciences, Xining, 810008 China; 5 BGI Institute of Applied Agriculture, BGI-Shenzhen, Shenzhen, 518120 China

**Keywords:** *Nitraria sibirica* Pall., Fruit color, Anthocyanin biosynthesis, MYB transcription factor, Tobacco

## Abstract

**Background:**

*Nitraria sibirica* Pall. is an economic plant with two kinds of fruit color, widely spreads in the Qinghai Tibet Plateau. The chemical analysis and pharmacological evaluation had been carried out for several tens of years, the mechanism behind the fruit color differentiation is still unclear.

**Results:**

In this manuscript, the chemical analysis of the extractions showed that the chemical composition of fruit color was anthocyanin, and two kind of *Nitraria sibirica* Pall. were caused by the content differentiation with the same anthocyanin kinds. Cyanidin-3-[2”-(6′”-coumaroyl)-glucosyl]-glucoside (C3G) was the major anthocyanin. Transcriptome analysis and the qRT-PCR revealed that the structural genes relative to anthocyanin biosynthesis except *CHS*, *F3’5’H* and *ANS* were up-regulated in the peels of BF (Black fruit) compared with the peels of RF (Red fruit), which indicated that transcript factor should be the reason for the expression difference of the structure genes. In the unigenes of the transcript factor MYB and bHLH, relative to anthocyanin, only *NsMYB1* (*Cluster 8422.10600*), was high-expression and up-expression in the peels of BF. *NsMYB1* encoded the same length protein with four amino acid differences in the RF and BF, and both contained the intact DNA, HTH-MYB and SANT domains. *NsMYB1* was close to the *AtMYB114*, *AtMYB113* and *AtPAP1*, regulating anthocyanin biosynthesis, in phylogenetic relationship. Both *NsMYB1r* and *NsMYB1b* could promote the transcript of the structural genes, and induced the anthocyanin accumulation in all tissues of transgenic tobacco. The insertion of ‘TATA’ in the promoter of *NsMYB1r* gave one more promoter region, and was the reason for higher transcripts in black fruit possibly.

**Conclusions:**

Cyanidin-3-[2′’-(6′”-coumaroy**l**)-glucosyl]-glucoside was the major anthocyanin in black fruit of *Nitraria sibirica* Pall.. *NsMYB1* was a functional R2R3-MYB transcription factor, regulated the anthocyanin biosynthesis, and led to the fruit color differentiation in *Nitraria sibirica* Pall.

**Supplementary Information:**

The online version contains supplementary material available at 10.1186/s12870-022-03561-5.

## Background

*Nitraria sibirica* Pall. belongs to the family *Nitrariaceae* Lindl [1], and is widespread in Central Asia, Kazakhstan, China, Mongolia, Siberia [2, 3]. This plant was branched halophyte with grayish-white bark, which can grow in the desert, saline and coastal saline-alkali lands [4]. *Nitraria sibirica* Pall. exhibits great adaptability to drought and saline-alkali soil, which can be used to reduce the soil salinization and used for windbreak and sand fixation [5]. Fruit of *Nitraria sibirica* Pall., also called desert cherry [6], is a fleshy drupe [2], which has been eaten as a functional food with the benefit for human nutrition and health on the Tibetan Plateau for thousands years. Hundreds of compounds have been identified from *Nitraria* fruits, which contained flavonoids, phenolic acids, alkaloids and so on. The pharmacological activities of *Nitraria* fruits have been studied extensively, including antioxidant activities in vitro and in vivo [7], anti-inflammatory activities [8, 9], neuroprotective effects [10] and α-glucosidase inhibitory effects [11]. Usually, the mature fruits of *Nitraria* showed the red color (RF) or black color (BF) (Purple color), but the chemical and genetic mechanism behind this color differentiation were unknown.

Anthocyanin is responsible for red, purple and blue coloration of flowers and fruits in plants [12]. Anthocyanin is water-soluble flavonoids, which mainly exists in cell vacuoles. Anthocyanin biosynthesis is a specific branch of the flavonoid synthesis pathway [13], which have been well studied in many plants, especially in model plants. A large number of structural genes involved in anthocyanin biosynthesis, which including phenylalanine ammoniacalyase (*PAL*), 4-coumaryl: CoA ligase (*4CL*), chalcone synthase (*CHS*), chalcone isomerase (*CHI*), flavonoid-3′-hydroxylase (*F3’H*), flavonoid-3′,5′-hydroxylase (*F3’5’H*), flavanone 3-hydroxylase (*F3H*), dihydro flavonol-4-reductase (*DFR*), anthocyanidin synthase (*ANS*), and flavonoid 3-O-glucosyltransferase (*UFGT*) [12, 14–16]. Generally, the structural genes of anthocyanin biosynthesis are regulated by transcription factors, WD40, bHLH and R2R3-MYB proteins [17–19]. The transcription factors regulate the expression of structural genes by forming trimer complexes and binding with the promoters of structural genes [20]. The allelic variations of the transcription factors were associated with the phenotype variation relative to anthocyanin biosynthesis. A lot of R2R3-MYB TFs had been identified as the key regulators of anthocyanin accumulation and the tissue coloration in some plants, such as *MiMYB1* in mango [21], *MdMYBA* and *MdMYB10* in apple [22, 23], *VvMYB114*, *VvMYB5b* and *VvMYBAs* in grape [24–26], *PcMYB114* in pear [27], *PaMYB10* in sweet cherry [28], *VcMYBA* in blueberry [29] and *PpMYB10.1* and *PpMYB10.3* in peach [30].

In this study, chemical analysis and RNA-seq were employed to understand the chemical and genetic basis for the fruit color differentiation in *Nitraria*. UPLC-MS was used to identify the chemical structure of the candidate key chemicals, and in vitro expression of the candidate key gene was carried out to validate its functions. The results showed that the anthocyanin content caused the color differentiation in *Nitraria*, and the MYB transcription factor *NsMYB1* was involved in the anthocyanin accumulation in *Nitraria*.

## Results

### The pigment isolation and identification in fruits of *Nitraria sibirica* Pall

**Fig. 1:**
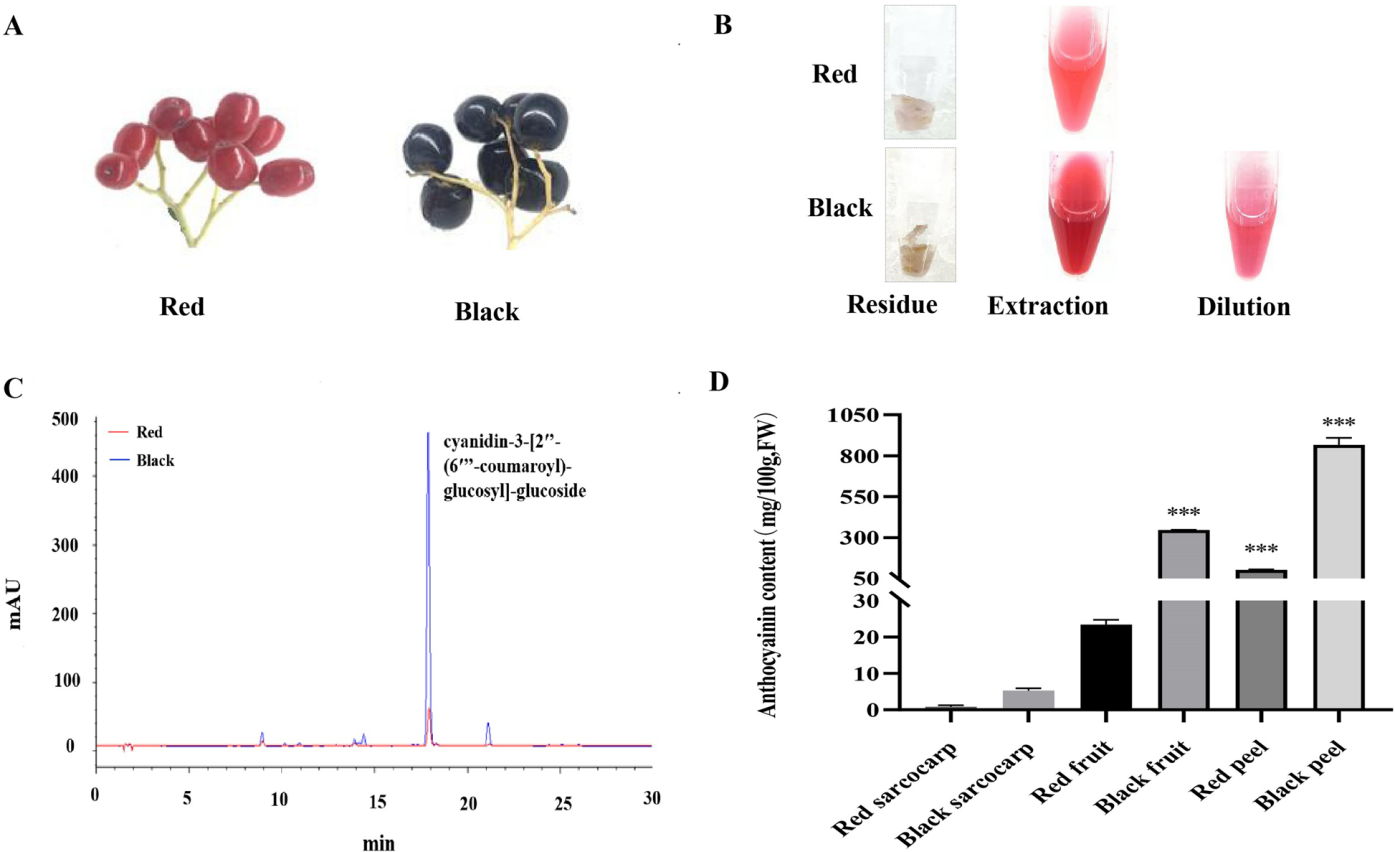
Anthocyanin content in the fruit of *Nitraria sibirica* Pall. **A** Photographs of *Nitraria sibirica* Pall. with RF (red fruit) and BF (black fruit). **B** Phenotype of the extract and residues after ultrasonic extraction. **C** The HPLC profiles (520 nm) of the anthocyanin from RF and BF of *Nitraria sibirica* Pall. **D** The total anthocyanin content in three fruit parts of RF and BF. Data represent means ± SD of three biological duplications. *, *p* < 0.05, **, *p* < 0.01, ***, *p* < 0.001

Obvious difference between RF and BF of *Nitraria sibirica* Pall. can be distinguished by naked eye (Fig. 1A). Apparently, the L*, a* and b* value of RF (31.15, 30.56 and 14.38) were all higher than BF (18.98, 0.93 and − 0.25). RF is brighter and greener than BF (Fig. S1). After ultrasonic extraction with methanol (1% HCl), the pigment could be extracted relatively thoroughly. The residues were nearly colorless (Fig. 1B). The extraction of BF was significantly darker than that of RF (Fig. 1B). The HPLC were employed to identify the compound responsible for the fruit color. Considering the color of the extraction was red, and the detection wavelength was chosen to be 520 nm. Seven kinds of the pigment compounds existed in both BF and RF with the content difference, and one compound was far higher compared with other compounds. When the extractions of BF were diluted 10 times, the color was light red, which closed to the extraction of RF (Fig. 1B). The HPLC analysis results revealed that RF and BF had the similar pigment chemicals, and only the content was higher in BF than RF (Fig. 1C). There were no special chemical compounds in BF.

The pigment composition in the BF of *Nitraria sibirica* Pall. was analyzed by the UPLC-TOF/MS. Seven anthocyanins were found in the extraction of BF (Fig. S2, Table 1). They were cyanidins, pelargonidins, and peonidin with different glucoside, coumaroyl, and caffeoyl. The highest content peak was identified as cyanidin-3-[2′’-(6′”-coumaroyl)-glucosyl]-glucoside (C3G) based on ESI-MS data. The domain anthocyanin peak had a molecular ion of m/z 757, MS fragments of m/z 287. The analysis of total ion flow charts (TIC) and MS/MS spectrums of anthocyanins were presented in the supplementary materials (Fig. S2), which was consistent with previous researches in *Nitraria* mature fruit [10, 31, 32]. Total anthocyanin content measurement showed that anthocyanins were mainly accumulated in the peels of fruits and the anthocyanin content in the BF was nearly 15 times as RF (Fig. 1D).

**Table 1:** Anthocyanins identified from the extract of *Nitraria sibirica* Pall (black fruit)

Peak	Anthocyanin	RT (min)	M+ (m/z)	MS fragments (m/z)
1	Cyanidin-3-O-[2-O-(β-Dglycopyranosyl)-β-Dglucopyranoside]	5.051	611	287
2	Cyanidin-3-O-sambubioside	5.456	581	287
3	Pelargonidin-3-O-diglucoside	5.597	595	271
4	Peonidin-3-O-diglucoside	6.287	625	301
5	cyanidin-3-[2″-(6′”-coumaroyl)-glucosyl]-glucoside	6.602	773	287
6	Cyanidin-3-[2″-(6′ “-coumaroyl)-glucosyl]-glucoside	7.678	757	287
7	Pelargonidin-3-[2″-(6′ “-transcoumaroyl)-glucosyl]-glucoside	8.740	741	271

### Transcriptomic analysis uncover the anthocyanin biosynthesis in RF and BF

**Fig. 2:**
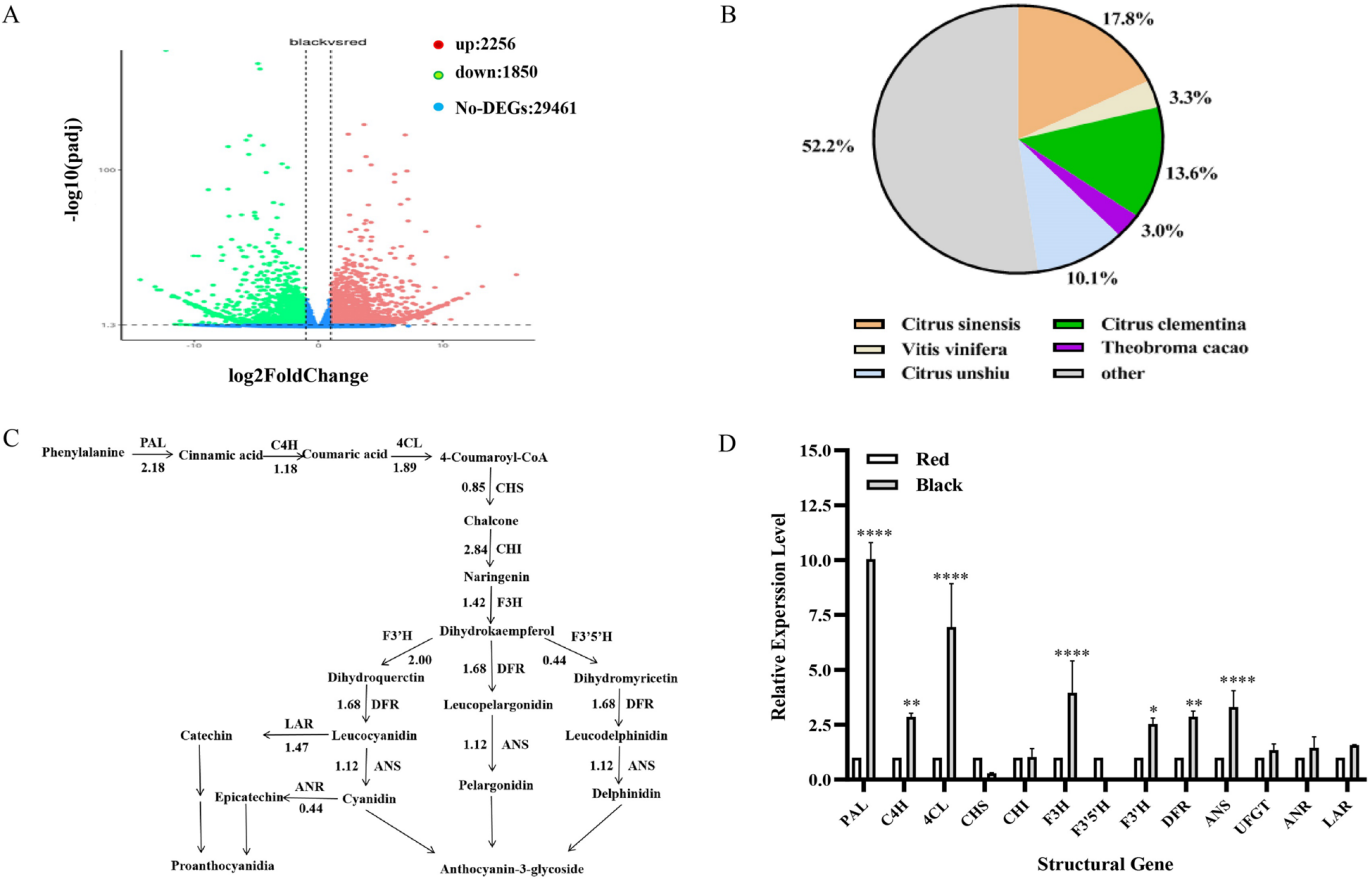
RNA-seq and qRT-PCR uncovered the transcriptome difference in RF and BF. **A** Differentially expressed genes in the peels of RF and BF. The genes were divided into three classes. The red dots indicated that the genes were up-regulated in the peels of BF. Green dots indicated genes that were down-regulated. Blue dots indicate that genes represent no DEGs. The X-axis represents the log2FoldChange. The Y-axis represents the Padj value. **B** The species classification of the unigenes of *Nitraria sibirica* Pall. **C** The expression differences of the structural genes in anthocyanin biosynthesis in the peels of RF and BF of *Nitraria sibirica* Pall. based on the RNA-seq. Arrow showed the metabolic stream, abbreviation left or upward arrows represent the genes catalyzing the progress, the number represent the average log2foldchange of the transcript level in the peels of BF against RF. **D** The relative expression level of thirteen structural genes involved in the anthocyanin synthesis pathway by qRT-PCR analysis. Data represent means ± SD of three biological duplications. *, *p* < 0.05, **, *p* < 0.01, ****, *p* < 0.0001

**Fig. 3:**
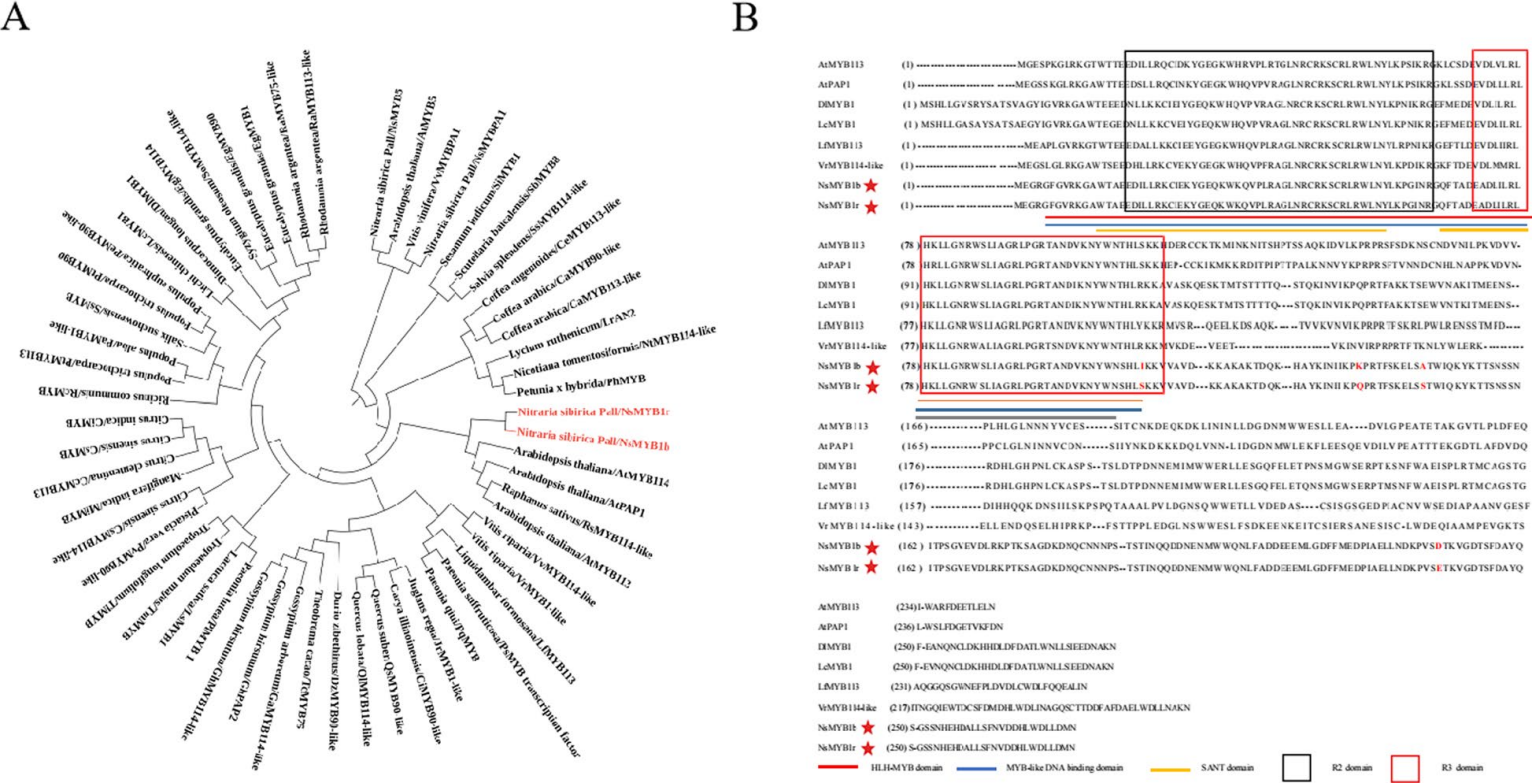
Phylogenetic tree and conservative domain analysis of *NsMYB1*. **A** Phylogenetic tree of *NsMYB1r*, *NsMYB1b* and anthocyanin-related R2R3-MYB transcription factors in other species. The accession number of these proteins follows the GenBank database: *AtPAP1*: OAP08844.1; *AtMYB114*: OAP11934.1; *AtMYB113*: NP_176812.1; *AtMYB5*: AAC49311.1; *VvMYBPA1*: NP_001268160; *LfMYB113*: AQM49950.1; *SsMYB*: NP_001233849; *CsMYB114-like*: XP_010511463.1; *NtMYB114-like*: NP_001306786.1; *PhMYB*:ADW94951.1; *LrAN2*: QCS14089.1; *TcMYB75*: XP_007033038.1; *SiMYB1*: XP_011099468; *SbMYB8*: AGZ16407.1; *DzMYB90-like*: XP_022728181.1; *GhPAP2*: AZT88307.1; *GhMYB114-like*: NP_001314544.1; *MiMYB*: BCB17000.1; *CsMYB114-like*: XP_006482384.1; *CcMYB113*: XP_024038103.1; *CsMYB*: NP_001275818.1; *CiMYB*: ANI87837.1; *LsMYB1*: XP_023769216.1; *CsMYB113*: XP_030492843.1; *PsMYB*: QIG55740.1; *PqMYB*: QCF29938.1; *PlMYB1*: QCF29938.1; *CeMYB113-like*: XP_027162525.1; *CaMYB90-like*: XP_027073233.1; *CaMYB113-like*: XP_027079539.1; *VvMYB114-like*: XP_034707784.1; *VrMYB1-like*: XP_034706167.1; *QsMYB90-like*: XP_023919955.1; *QlMYB114-like*: XP_030952856.1; *CiMYB90-like*: KAG6720488.1; *JrMYB1-like*: XP_018816575.1; *PvMYB90-like*: XP_031273872.1; *PtMYB113*: XP_024444016.1; *PtMYB90*: XP_024444002.1; *PeMYB90-like*: XP_011021394.1; *RsMYB*: AVI16683.1; *RnMYB*: AVI16682.1; *TmMYB*: QTO65864.1; *TlMYB*: QTO65862.1; *LcMYB*: APP94121.1; *DlMYB1*: QRM13298.1; *EgMYB114*: XP_010062250.2; *SoMYB114-like*: XP_030461286.1; *EgMYB1*: XP_010062250.2; *EgMYB90*: XP_010064837.2; *RaMYB75-like*: XP_030525682.1; *RaMYB113-like*: XP_030537747.1. **B** The alignment of the amino acid sequences of *NsMYB1b*, *NsMYB1r* and the MYB proteins relative to anthocyanin biosynthesis. Numbers indicated the position of the last amino acid in each line of the proteins. The amino acid differences of *NsMYB1b*, *NsMYB1r* were indicated with red star. Different color lines represent HTH_MYB, MYB-like DNA-binding and SANT domain. Black and red frames indicate R2 domain and R3 domain, respectivly. The GenBank accession numbers were as follows: *AtMYB113*: OAP11934.1; *AtPAP1*: AAG42001; *DlMYB1*: QRM13298.1; *LcMYB1*: APP94121.1; *LfMYB113*: QVX18575.1; *Vr MYB114*: XP_034711250.1

**Fig. 4:**
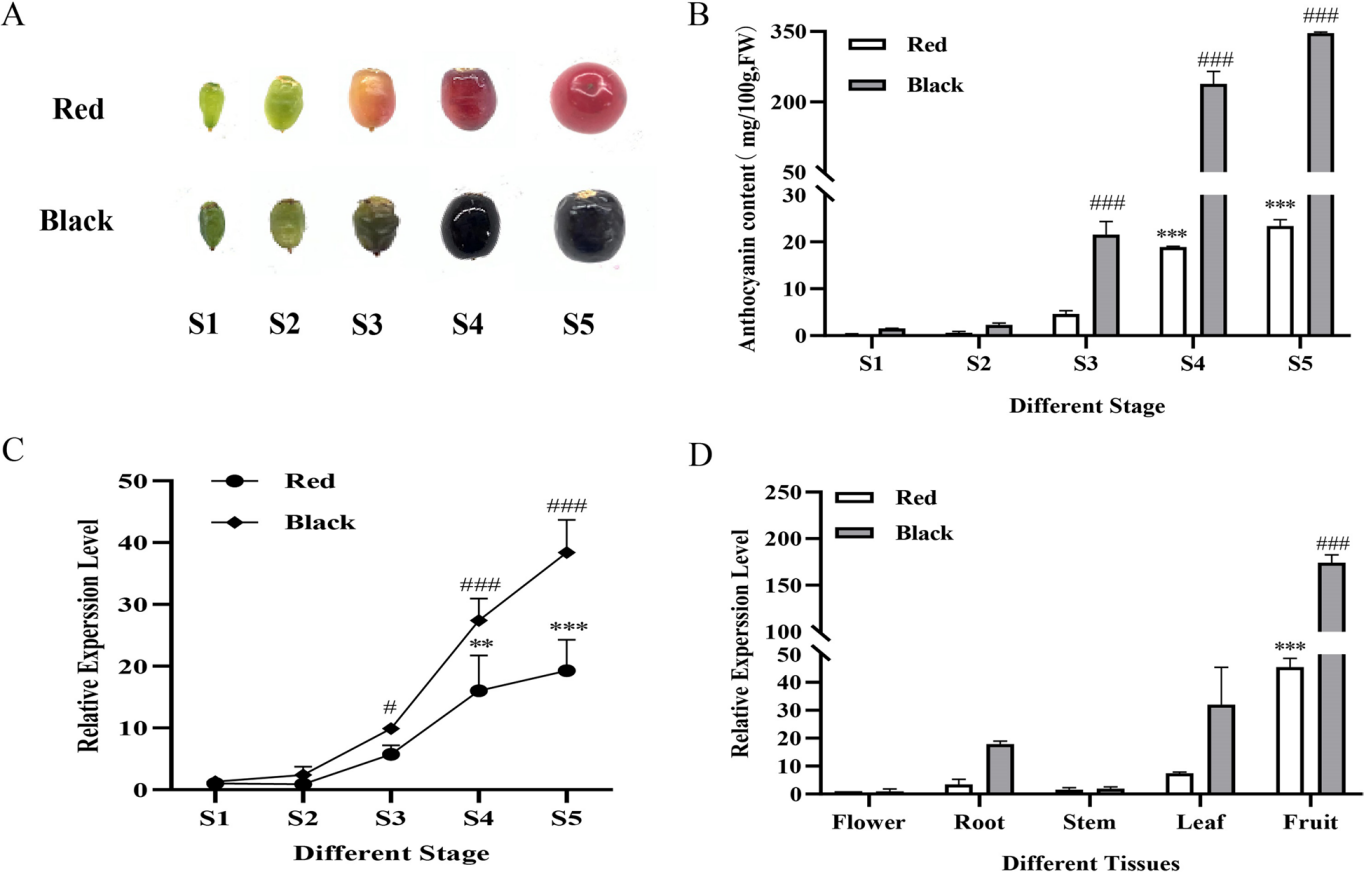
The total anthocyanin content in different stages and the expression profiles of *NsMYB1* in *Nitraria sibirica* Pall. **A** Fruits at five developmental stages. **B** The total anthocyanin content of the fruits at five developmental stages. **C** The relative expression level of *NsMYB1* at five developmental stages. **D** The relative expression level of *NsMYB1* in different tissues. Data represent means ± SD of three biological duplications. * or #, *p* < 0.05, ** or ##, *p* < 0.01, ***or ###, *p* < 0.001

Because the color differentiation was caused by the anthocyanin content, transcriptomic analysis was employed to uncover the gene expression difference in the peels of RF and BF. In total, 22,427,135 and 23,236,348 raw reads were obtained from the peels of RF and BF cDNA library. After filtering, 21,638,393 and 22,187,516 clean reads were obtained with Q20 at 97.60% and Q30 at 93.29%, the GC percentage were 44.9 and 44.6% (Table S2). 33,567 unigenes were obtained based on Trinity software. After the FPKM value evaluation, in contrast with the peels of RF, 2256 unigenes were up-regulated and 1850 unigenes were down-regulated in the peels of BF (Fig. 2A). 199 DEGs can be classified to the pathway biosynthesis secondary metabolite in KEGG pathway (Fig. S3). Homology comparison showed the predicted proteins of the peels of RF and BF had high homology with *citrus sinensis* (17.8%), *citrus clementine* (13.6%) and *cirtus unshiu* (10.1%) (Fig. 2B). Because the color difference of RF and BF depended on the anthocyanin accumulation, thirteen structural genes relative to anthocyanin biosynthesis were selected for TBLASTN search. Except for *F3’5’H*, *CHS* and *ANR*, other structural genes were up-expressed in the peels of BF with between 1.18 and 2.8 times (Fig. 2C). The up-expression of the structural genes indicated that transcription factor was the key gene for the color differentiation. Two unigenes *Cluster-8422.11741* and *Cluster-8422.1914* were homologous to bHLH transcription factors with *AtJAF13* as reference sequence. In phylogenetic tree, *Cluster-8422.11741* and *Cluster-8422.1914* were close to *AtTT8* and *AtJAF13*. The FPKM value of *Cluster-8422.11741* and *Cluster-8422.1914* were 4263.45 and 55.36 in the peels of BF, respectively. The *cluster-8422.11741* should be the bHLH transcription factor, involving in anthocyanin biosynthesis (Tables S3, S5). The Log2FoldChange of *cluster-8422.11741* were − 1.08, which meant the expression of *cluster-8422.11741* was higher in the peels of RF than BF. Three unigenes were homologous to the MYB transcription factor with *LbAN2* as reference sequence. In phylogenetic tree, *Cluster-8422.761* and *Cluster-8422.21912* were mainly related to procyanidins synthesis. The *Cluster-8422.10600* was relative to anthocyanin biosynthesis (Tables S3, S5, Fig. 3A). The FPKM value of *Cluster-8422.761*, *Cluster-8422.21912* and *Cluster-8422.10600* were 222.12, 172.03, 2832.66, respectively. The transcript level of *Cluster-8422.10600* was far higher than other unigenes. *Cluster-8422.10600* should be the main MYB transcription factor, involving in anthocyanin biosynthesis. The Log2FoldChange of *Cluster-8422.10600* was 0.52, which meant the expression of *Cluster-8422.10600* was higher in the peels of BF than RF. Based on comprehensive consideration of homology, FPKM and Log2FoldChange, the *Cluster-8422.10600* should be the candidate key gene for the color differentiation in *Nitraria sibirica* Pall. The qRT-PCR also conformed the relative transcript level of the structural genes and the candidate key gene *Cluster-8422.10600* (Figs. 2D and 4B). *Cluster-8422.10600* was named as *NsMYB1* for the further analysis.

### Isolation and characterization of *NsMYB1* from *Nitraria sibirica* Pall

The genomic DNA (gDNA) and cDNA sequences of *NsMYB1r* and *NsMYB1b* were isolated from RF and BF of the *Nitraria sibirica* Pall. based on RNA-sequence data. The gDNA were 3471 and 3472 bp length, respectively. Both *NsMYB1r* and *NsMYB1b* had three exons and two introns. Open reading frames (ORFs) of *NsMYB1r* and *NsMYB1b* were 831 bp length and encodes a protein of 276 amino acid. Four nucleotide sequence differences caused four amino acid sequence differences between *NsMYB1r* and *NsMYB1b* (Fig. 3B, Fig. S4). According to SOPMA prediction results, the main structure of *NsMYB1r* and *NsMYB1b* was random coil, which were 59.78 and 57.25%, the second was alpha helix, which were 30.43 and 33.33%. *NsMYB1r* and *NsMYB1b* were closest to *AtPAP1*, *RsMYB114-like*, *AtMYB113* and *AtMYB114* in the phylogenetic tree (Fig. 3A). All genes in these cluster were relative to the anthocyanin biosynthesis, such as *AtPAP1* (*Arabidopsis thaliana*), *SlMYB75* (*Solanum lycopersicum*), *BrMYB75* (*Brassica rapasubsp*), *VvMYB114* (*Vitis vinifera*) and *VrMYB114* (*Vitis riparia*). *NsMYB5* (*Cluster-8422.761*) and *NsMYBPA1* (*Cluster-8422.21912*) were closest to *AtMYB5* and *VvMYBPA1* (Fig. 3A), which were mainly contribute to the procyanidins accumulation. Compared with *AtMYB113*, *AtPAP1*, *DlMYB1*, *LcMYB1*, *LfMYB113*, *VrMYB114*, both *NsMYB1r* and *NsMYB1b* contained the complete MYB-like DNA-binding, SANT domains and HTH-MYB domain (Fig. 3B). In the four amino acids differences in *NsMYB1r* and *NsMYB1b*, only S > I existed in the R3 domain.

The promoters were isolated from *NsMYB1* by TAIL-PCR. The promoters of *NsMYB1r* and *NsMYB1b* were 894 and 899 bp length (Fig. S5). Compared to the promoter of *NsMYB1r*, ‘TATA’ sequences were inserted in the promoter region from -221 bp to − 218 bp of *NsMYB1b* (Figs. S5, S6). The promoter prediction based on the software BDPG showed that the promoter from *NsMYB1b* had three possible promoter regions, while *NsMYB1r* only contained two. The ‘TATA’ sequence gave *NsMYB1b* one unique promoter (Table S4), which may promote *NsMYB1b* expression to active anthocyanin synthesis pathway in the black phenotype.

### Expression of *NsMYB1* correlates with anthocyanin biosynthesis

The total anthocyanins content and the expression pattern of *NsMYB1* were measured at five development stages (5, 25, 45, 65 and 85 DAF). The total anthocyanin content with five development stages showed that anthocyanin accumulation mainly started from 45 DAF (S3, Color Changing Period) and reached the highest level in 85 DAF (S5, mature stage). The anthocyanin content in BF was always higher than that in RF in every stage (Fig. 4A, B). In all stages, the expression levels of *NsMYB1* were higher in BF than RF (Fig. 4C). The relative expression level of the *NsMYB1* was consistent with anthocyanin accumulation. The anthocyanin content in fruit was much higher than the flower, leaf, root and stem in version. (Fig. S7). The relative expression level of *NsMYB1* in fruit was far higher than other tissues, and *NsMYB1* was higher expressed in these tissues of BF than RF (Fig. 4D). These results strongly suggested that *NsMYB1* expression was correlated to the anthocyanin accumulation in *Nitraria sibirica* Pall.

### Overexpression of *NsMYB1* induced the anthocyanin accumulation in tobacco

**Fig. 5:**
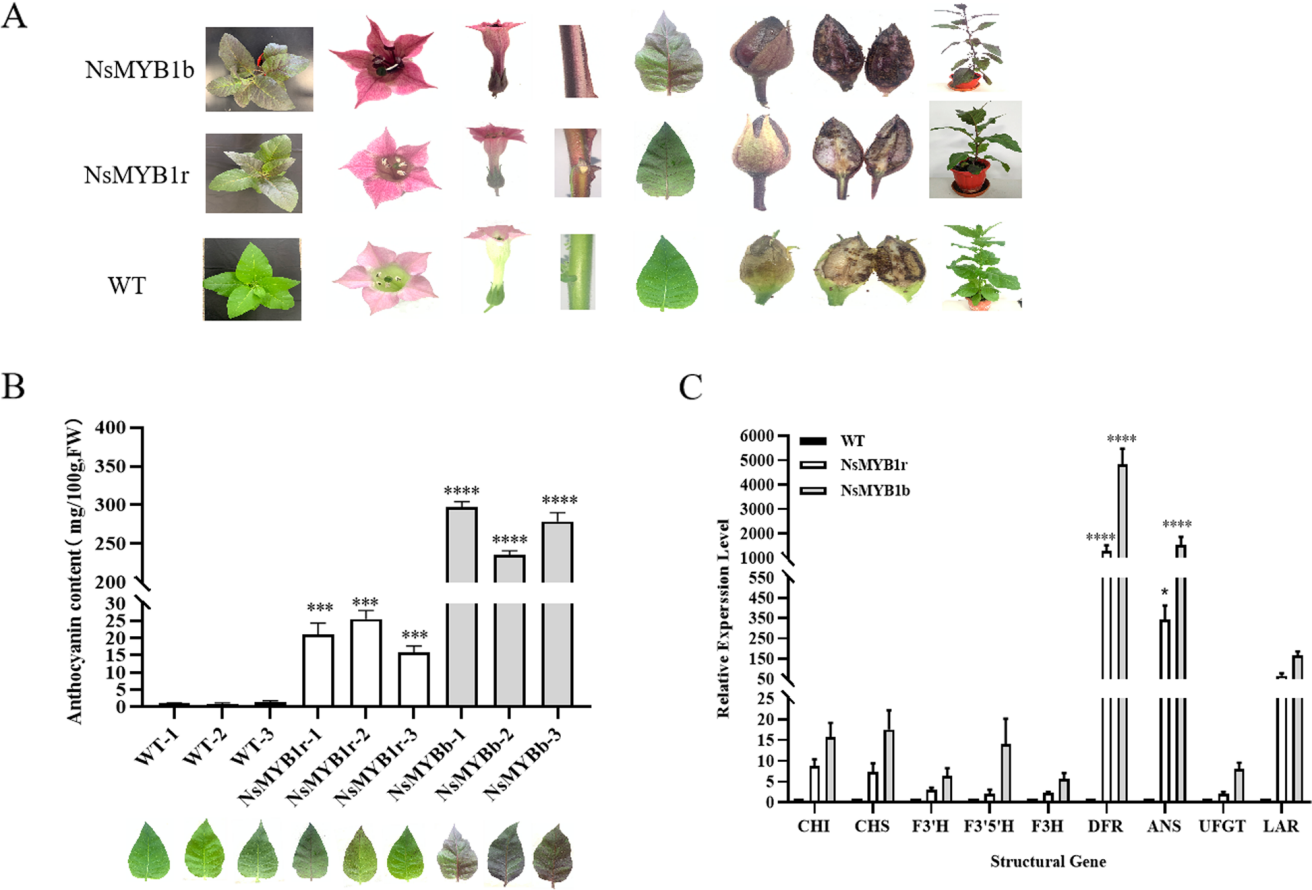
Phenotype, the total anthocyanin content and the relative expression level of structural genes between WT and transgenic tobacco. **A** Phenotype of transgenic lines and WT. WT, wild type plant. **B** The total anthocyanin content of transgenic lines. **C** The relative expression level of nine structural genes associated with anthocyanin synthesis in WT, *NsMYB1r* and *NsMYB1b* transgenic tobacco. Data represent means ± SD of three biological duplications. *, *p* < 0.05, ***, *p* < 0.001, ****, *p* < 0.0001

For investigating the actual function, *NsMYB1r* and *NsMYB1b* were overexpressed in *Nicotiana tabacum*. Almost all tissues of the transgenic lines showed be stained with higher anthocyanin content compared with WT. The transgenic lines of *NsMYB1b* had the dark purple phenotype, while transgenic lines of *NsMYB1r* presented light-purpled plant organs (Fig. 5A). The total anthocyanin content of the root, stem, leaf and flower of the *NsMYB1b* transgenic lines were higher than that of *NsMYB1r* transgenic lines (Fig. 5B, Fig. S8A). The anthocyanin was mainly accumulated in flower and leaf in transgenic lines. The qRT-PCR results showed that the relative expression level of *NsMYB1* and the anthocyanin synthesis-related structural genes, *NtCHI*, *NtCHS*, *NtF3’H*, *NtF3’5’H*, *NtF3H*, *NtDFR*, *NtANS*, *NtUFGT* and *NtLAR* were all up-regulated in the transgenic lines of *NsMYB1b* and *NsMYB1r* (Fig. 5C, Fig. S8B). *NtDFR* had the highest differential expression level. These results suggested that *NsMYB1* was a functional MYB transcription factor regulating anthocyanin biosynthesis.

## Discussion

In this study, we focused on the chemical and genetic mechanism of the differentiation of the fruit color of *Nitraria sibirica* Pall. with red and black phenotype. We extracted and identified the chemical compounds responsible for the red and black fruit of *Nitraria sibirica* Pall.. Two R2R3 MYB transcription factors genes *NsMYB1r* and *NsMYB1b* were isolated and functional verified from *Nitraria sibirica* Pall. The relationship between anthocyanin synthesis and the phenotype differentiation of the fruit was discussed.

### The anthocyanin content should be the reason for the color differentiation of *Nitraria sibirica* Pall

The color differentiation of the fruits of *Nitraria sibirica* Pall. should be derived from the different anthocyanin accumulation. The pigment compounds were easily extracted from the methanol solution (1% HCl, v/v), which was the typical characteristic of anthocyanin. The plant pigment carotene can’t be easily extracted in the same condition. The pigment compounds in the extraction could be detected in 520 nm detection wave length, which were responsible for the red color in visible light spectrum. The kinds of the pigment compound were similar in the BF and RF with one main peak. After the dilution, the extraction color of BF was similar to the extraction of BF. The UPLC-TOF/MS identified seven pigment compounds in BF extraction in the red color detection wave, and all of them were anthocyanins with different structures. It could be inferred that the anthocyanin content should be the reason for the color differentiation in the BF and RF. Actually, the anthocyanin contents in the BF were almost 15 times of the RF.

### *NsMYB1* is a functional MYB transcription factor gene regulating anthocyanin biosynthesis

Transcriptome and qRT-PCR showed that the structural genes relative to anthocyanin biosynthesis, except for *F3’5’H*, *CHS* and *ANR*, showed higher expression in the BF than RF. *F3H* was responsible for synthesizing all anthocyanin biosynthesis. *F3’H* was the key genes for cyanidins, and *F3’5’H* was the key gene for synthesizing delphinidin [33–35]. In the fruit of *Nitraria sibiric*a Pall., only cyanidin, pelargonidin, and peonidin could be detected, and the delphinidin didn’t existed. It could be explained that *F3’5’H* had no different expression in BF and RF, while *F3H* and *F3’H* had higher expression in BF than RF. The Log2FoldChanges of the genes relative to anthocyanin biosynthesis were relatively low compared with previous researches. Previous researches usually compared the materials with anthocyanin and no anthocyanin [36–38]. In this case, both materials could accumulate the anthocyanins. Usually, the transcript or structural difference of the transcription factor could induce the different expression of all structural genes. Based on the transcript level, one MYB transcription factor *NsMYB1* was chosen for further analysis.

Both *NsMYB1b* and *NsMYB1r* contained the MYB-like DNA binding, HTH-MYB and SANT domain, which were necessary for anthocyanin synthesis [39]. Phylogenetic tree demonstrated that *NsMYB1* belonged to the branch of R2R3 MYB transcription factors (*AtMYB114*, *AtMYB113* and *AtPAP1*). They are all relative to anthocyanin biosynthesis. Overexpression of *AtMYB113* or *AtMYB114* can result in substantial increases in pigment production [40], and the overexpression of *AtPAP1* resulted in enhanced accumulation of anthocyanin pigments in *Solanum nigrum* Lin. (Black Nightshade) [41]. In this case, overexpression of *NsMYB1r* and *NsMYB1b* induced the up-expression of the structural genes relative to anthocyanin biosynthesis, and the anthocyanin accumulation in all tissues of tobacco. *NsMYB1r* and *NsMYB1b* should be the functional R2R3 MYB transcription factors.

*NsMYB1* was only high-expression of MYB transcription factor regulating anthocyanin biosynthesis, and had higher expression in the peels of BF. The expression level of *NsMYB1* in BF and RF increased continuously with the anthocyanin accumulation during fruit ripening. The promoter difference should produce the higher expression in BF. The higher expression level of *NsMYB1b* may cause higher anthocyanin accumulation in black fruit of *Nitraria sibirica* Pall.

## Conclusion

In this manuscript, cyanidin derivatives are responsible for the black color pigments of *Nitraria sibirica* Pall fruits. Cyanidin-3-[2′’-(6′”-coumaroyl)-glucosyl]-glucoside was the major anthocyanin in black fruit of *Nitraria sibirica* Pall.. Functional R2R3-MYB transcription factor *NsMYB1* confirmed the main reason for high anthocyanin content in fruits, and the allelic variation of *NsMYB1* led to different color of fruits. This results would help us to understand the molecular regulatory mechanisms of anthocyanin biosynthetic pathway in the fruits of *Nitraria sibirica* Pall..

## Methods

### Plant materials

The red and black fresh fruits of *Nitraria sibirica* Pall. were picked from Zongjia Town, Dulan country in the Qaidam Basin, Qinghai Province (Longitude: 96^°^27.2856′E, Latitude: 36^°^26.9625′ N, Altitude: 2856.6 m) and identified by Qingbo Gao (Northwest Institute of Plateau Biology, Chinese Academy of Science). The voucher specimen (Nwipb0334878 and 0334879) were kept in the Herbarium of Northwest Institute of Plateau Biology, Xining, Qinghai Province. No permission was required in collecting the plants. The samples were harvested 5, 25, 45, 65 and 85 days after anthesis, the fruit peels were peeled from fresh fruits immediately, all samples were frozen in liquid nitrogen and then stored at − 80 °C until use. Three colour difference indexes (L*: brightness, black and white, a*: red-green, b*: yellow-blue) of the fresh fruits of *Nitraria sibirica* Pall. were measured by a CS-412 colorimeter (Hangzhou CHNSpec Technology Co., Ltd., Hangzhou, China). Fruits were picked from three individual plants, and three biological replicates were performed.

### HPLC/DAD and UPLC-ESI/MS analysis

1 g fresh fruits were extracted in 20 mL methanol containing 1% (v/v) hydrochloric acid. Then, ultrasound at 40 °C for 30 min, followed centrifugation at 4000 r/min for 10 min. After filtered through a 0.22 μm filter and retained for component analysis. The samples were analyzed by Agilent HPLC system (Agilent Technologies, USA). ZORBAX-SB C18 column (100 mm × 4.6 mm i.d., 5um, Agilent, USA) was used with the mobile phase of 0.1% trifluoroacetic acid-0.1% trifluoroacetic in acetonitrile by gradient elution. The applied gradient program was: 0 to 30 min, linear gradient from 10 to 30% B. The flow rate was 1 mL/min, and the temperature was 35 °C, the injection volume was 5 μL, the detection wavelength was 520 nm for identifying the pigment compounds.

In order to identify the chemical component of the pigment in fruit extract, UPLC-Triple-TOF/MS analysis method was applied. The sample was separated by ACQUITY UPLC HSS sb-C18 column (100 mm × 2.1 mm i.d., 1.7 μm). 1% formic acid solution as mobile phase A, 1% formic acid acetonitrile as mobile phase B, linear gradient elution. Specific elution procedure was set as follow: 0–5 min, 5–15% B, 5–12 min, 15–25% B, 12–20 min, 25–60% B, 20–23 min, 60–100% B. The flow rate was 0.3 mL/min, the column temperature was 50 °C, the detection wavelength was 520 nm and the injection volume was 3 μL. The peaks were further identified by ESI-MS. Positive ion scanning mode was selected for mass spectrometry (MS) over the rage m/z 100–1500. For the first order scanning, declustering potential (DP) and focusing voltage (CE) was 100 V and 10 V, respectively. For the second order scanning, mass spectrometry data were collected using TOF MS-Product Ion-IDA mode. All these pigment compounds were anthocyanin.

### Anthocyanin content determination

The anthocyanin was extracted from the fruits and the peels of fruits of *Nitraria sibirica* Pall with methanol (1% HCl, v/v). The total anthocyanin content was determined by using the pH-differential method [10, 42], with three repetitions in each plant. The absorbance of the sample of 525 nm and 700 nm were measured by using UV-vis spectrophotometer at pH 1.0 and pH 4.5. The total anthocyanin content was measured in terms of cyanidin-3-glucoside equivalent.

$$\mathrm{Anthocyanin}\;\mathrm{content}\;\left(\mathrm{cyanidin}-3-\mathrm{glucoside},\mathrm{mg}/100\mathrm g\right)=\frac{\mathrm A\times\mathrm{MW}\times\mathrm{DF}\times DV\times1000\times100}{\varepsilon\times W\times1}$$

Among, A = (A525 nm – A700 nm) pH 1.0 – (A525 nm – A700 nm) pH 4.5, MW is the molar mass of cyanidin-3-glucoside (449.2 g/mol), DF is dilution factor, W is the weight of sample (g), DV is the total value (mL), ε is the extinction coefficient for cyanidin 3-glucoside (26, 900 L/mol/cm).

### DNA, RNA isolation and cDNA synthesis

DNA was extracted by DNAprep Pure Plant Kit (Tiangen Company, Beijing, China). Total RNA was isolated by RNAprep Pure Plant Kit (Tiangen Company, Beijing, China). The quality of the total RNA and DNA were evaluated by 1% agarose gels and the purified concentrations were measured by NanoDrop (Thermo Scientific, Wilmington, DE, USA). First-strand cDNA was synthesized from total RNA for RT-PCR and RNA-seq by PrimeScript™ II 1st Strand cDNA Synthesis Kit (TaKaRa Code No.6210A). The first-strand cDNA was synthesized from total RNA for qRT-PCR using the Primer ScriptTMRT Master Mix (Perfect Real Time) (TaKaRa).

### RNA-Seq

The cDNA libraries were sequenced using the Illumina HiSeq 2000 (Illumina, San Diego, CA, USA), with three repetitions. The original sequencing results were filtering to remove joint sequences, low quality sequence, and reads containing poly-A, so as to obtain high quality sequences before data assembly. Then reliable transcripts were obtained by assembling high-quality data from sequencing, which using Trinity, a short-read assembly program [43]. Gene function was annotated using the following: the NCBI non-redundant (Nr), Swiss-Prot, the kyoto Encyclopedia of Gene and Genome (KEGG), Clusters of Orthologous Groups of proteins (COG), and the Gene Ontology (GO) database.

The expression level of the peels of RF and BF of *Nitraria sibirica* Pall were estimated by FPKM (expected number of Fragments Per Kilobase of transcript sequence per Millions base pairs sequenced) [44]. The differences in unigenes between the peels of RF and BF of *Nitraria sibirica* Pall. were analyzed by IDEG6 software (BGI, ShenZhen, GuangDong, China) [45]. The threshold for significantly differential expression was *P*-value< 0.05, and |log2 fold change| > 1 according to DESeq between two different cDNA libraries. GO and KEGG enrichment analysis of DEGS were using the R platform [46].

### qRT-PCR validation

The primers for the selected genes were designed by Primer 5.0 (Table S1). The qRT-PCR was conducted with the SYBR Premix Ex Taq TM II (Tli RNaseH Plus) (TaKaRa Code No. RR820A) in Applied Biosystems Quant Studio (Thermo Fisher Company, Beijing, China). The reaction system and procedure of qRT-PCR were completed by referring to previous literatures [47, 48]. The relative expression level was calculated by 2^-∆∆CT^ method. Three biological replicates were performed.

### Gene clone and construct expression vectors

The 50 μL reaction system contain 25 μL PrimeSTAR Max Premix (2×), 0.5 μL each primer, 23 μL ddH_2_O, and 1 μL DNA and cDNA (TaKaRa Code No. R045A). Primer sequences were designed to amplify the ORFs, which were listed in Table S1. The cycling conditions were as follows: 30 cycle at 98 °C for 10 s, 55 °C for 5 or 15 s and 72 °C for 1 min. PCR fragments were extracted with the Tiangen TIANgel Midi Purification Kit (Tiangen) from 1.0% agarose gels and were cloned into the pEASY®-Blunt vector (TransGen Biotech, Beijing, China), which transformed into *Escherichia coli*. DH5α cell, then, the positive cloned were sequenced by sangon (Shanghai, China).

The overexpression vectors of *NsMYB1r* and *NsMYB1b* were constructed with vector PC2300s by double-digested using restriction enzymes of SacI, BamHI (TaKaRa). Then the PC2300s:NsMYB1r and PC2300s:NsMYB1b recombinant vectors were transformed into *Agrobacterium tumefaciens* LBA4404.

### Overexpression of *NsMYB1* in tobacco

The leaf disc transformation method was used for tobacco transformation [49]. The selective media component of tobacco as follows: 0.44% (w/v) agar, 0.44% (w/v) Vitamin, 3% (w/v) sucrose, 0.1 mg/L 1-Naphthaleneacetic acid (NAA), 1 mg/ L 6-benzylaminopurine (BAP), 10 mg/L kanamycin, and 10 mg/L Cefotaxime. The transgenic shoots grow up in the incubator with long-day lighting (16 h light/8 h dark), the nutrient media need periodic replacement.

### Isolation of *NsMYB1* promoter region

The promoter sequences of *NsMYB1* were isolated from RF and BF by Thermal asymmetric interlaced polymerase chain reaction (TAIL-PCR) [50]. The functional domain in *NsMYB1* promoter sequences were analyzed based on BDPG (http://www.fruitfly.org/seqtools/promoter.html) [51].

### Bioinformatics analysis

The website (http://www.ebi.ac.uk/interpro/) was used to predict the conservative functional domains. Phylogenetic tree of *NsMYB1* was constructed by MEGA 5.1 with neighbor-joining phylogeny testing and 1000 boot strap replicates. Vector NTI 10 software (Thermo Fisher Scientific) was used to sequence alignments. KEGG enrichment analysis of DEGS was used the website (www.kegg.jp/kegg/kegg1.html). The secondary structure was predicted by SOPMA secondary structure prediction method [52].

### Statistical analysis

Statistical analysis was conducted using GraphPad Prism 8.0 software (GraphPad Software, Inc., USA). Significant differences were depended on Student’s t-test and one-way ANOVA. Differences with *p*-values < 0.05 were considered significant. All data was showed as means ± SD.

## Supplementary Information

**Additional file 1.**

## Data Availability

The transcriptomic data has been successfully uploaded to NCBI (http://www.ncbi.nlm.nih.gov/bioproject/788651), Submission ID: SUB10797457; BioProject ID: PRJNA788651. All data generated or analyzed during this study are included within the article and its additional files.

## Abbreviations

PAL: Phenylalanine ammoniacalyase
4CL: 4-coumaryl: CoA ligase
CHS: Chalcone synthase
CHI: Chalcone isomerase
F3H: Flavonoid-3-hydroxylase
F3’H: Flavonoid − 3′- hydroxylase
F3’5’H: Flavonoid-3′, 5′-hydroxylase
DFR: Dihydroflavonol 4-reductase
ANS: Anthocyanidin synthase
UFGT: Flavonoid3-O-glucosyltransferase
CDS: Coding sequences
WT: Wild type
bHLH: Basic Helix-Loop-Helix
qRT-PCR: Quantitative real-time polymerase chain reaction
